# Association between Pulmonary Aspergillosis and *Cytomegalovirus* Reactivation in Critically Ill COVID-19 Patients: A Prospective Observational Cohort Study

**DOI:** 10.3390/v15112260

**Published:** 2023-11-15

**Authors:** Valeria Caciagli, Irene Coloretti, Marta Talamonti, Carlotta Farinelli, Ilenia Gatto, Emanuela Biagioni, Mario Sarti, Erica Franceschini, Marianna Meschiari, Cristina Mussini, Roberto Tonelli, Enrico Clini, Massimo Girardis, Stefano Busani, Modena COVID-19 Working Group

**Affiliations:** 1 Anesthesia and Intensive Care Medicine, Policlinico di Modena, University of Modena and Reggio Emilia, 41124 Modena, Italyemanuela.biagioni@gmail.com (E.B.);; 2 Microbiology and Virology Unit, Azienda Ospedaliero-Universitaria Policlinico, 41124 Modena, Italy; 3 Infectious Diseases Unit, Policlinico di Modena, University of Modena and Reggio Emilia, 41124 Modena, Italymariannameschiari1209@gmail.com (M.M.);; 4 Respiratory Diseases Unit, Policlinico di Modena, University of Modena and Reggio Emilia, 41124 Modena, Italy

**Keywords:** COVID-19, invasive pulmonary aspergillosis, critically ill, *cytomegalovirus*, ards

## Abstract

COVID-19-associated invasive pulmonary aspergillosis (CAPA) is common and is associated with poor outcomes in critically ill patients. This prospective observational study aimed to explore the association between CAPA development and the incidence and prognosis of *cytomegalovirus* (CMV) reactivation in critically ill COVID-19 patients. We included all consecutive critically ill adult patients with confirmed COVID-19 infection who were admitted to three COVID-19 intensive care units (ICUs) in an Italian hospital from 25 February 2020 to 8 May 2022. A standardized procedure was employed for early detection of CAPA. Risk factors associated with CAPA and CMV reactivation and the association between CMV recurrence and mortality were estimated using adjusted Cox proportional hazard regression models. CAPA occurred in 96 patients (16.6%) of the 579 patients analyzed. Among the CAPA population, 40 (41.7%) patients developed CMV blood reactivation with a median time of 18 days (IQR 7–27). The CAPA+CMV group did not exhibit a significantly higher 90-day mortality rate (62.5% vs. 48.2%) than the CAPA alone group (*p* = 0.166). The CAPA+CMV group had a longer ICU stay, fewer ventilation-free days, and a higher rate of secondary bacterial infections than the control group of CAPA alone. In the CAPA population, prior immunosuppression was the only independent risk factor for CMV reactivation (HR 2.33, 95% C.I. 1.21–4.48, *p* = 0.011). In critically ill COVID-19 patients, CMV reactivation is common in those with a previous CAPA diagnosis. Basal immunosuppression before COVID-19 appeared to be the primary independent variable affecting CMV reactivation in patients with CAPA. Furthermore, the association of CAPA+CMV versus CAPA alone appears to impact ICU length of stay and secondary bacterial infections but not mortality.

## 1. Introduction

Invasive pulmonary aspergillosis (IPA) primarily affects immunocompromised patients, including those with severe and prolonged neutropenia, hematological malignancies, organ transplant recipients, and individuals with structural lung damage receiving systemic corticosteroids [1]. In the last few decades, several cases of IPA have been described in ICU patients hospitalized for influenza pneumonia among critically ill patients without immunosuppression, and severe influenza is now recognized as a risk factor for IPA [2]. Recently, severe COVID-19 pneumonia has emerged as a risk factor, leading to the recognition of COVID-19-associated pulmonary aspergillosis (CAPA) as a significant complication among critically ill COVID-19 patients [3]. Approximately 10–20% of ICU-admitted COVID-19 patients eventually develop CAPA [4,5], which is associated with high mortality rates [5].

The relationship between *cytomegalovirus* (CMV) and IPA is well established in immunocompromised individuals [6] but has been less explored in critically ill patients [7], especially those with COVID-19 [8].

This observational study aimed to describe the link between CMV replication and CAPA occurrence in COVID-19 patients admitted to our ICUs.

## 2. Materials and Methods

This observational study analyzed prospectively collected data from all consecutive adult patients admitted to the three COVID-19 ICUs at the University Hospital of Modena with confirmed SARS-CoV-2 infection and moderate to severe acute respiratory distress syndrome (ARDS) from 25 February 2020 to 8 May 2022 [9]. Patients who were aged < 18 years who had an ICU length of stay (LOS) < 24 h, limitation of care, or lack of resuscitation order were excluded from the analysis. This study was approved by the Institutional Ethics Committee of Area Vasta Emilia Nord (approval number:396/2020/OSS/AOUMO—CoV-2 MO-Study). Owing to the observational nature of this study, written informed consent was not required.

### 2.1. Treatment Protocol

All patients received standard ICU and supportive care as recommended by the World Health Organization (WHO) guidelines [10], specific therapies according to national guidelines [11], and local protocols for COVID-19 treatment, including dexamethasone and low-molecular-weight heparin for prophylaxis of deep vein thrombosis according to individual body weight and renal function. In addition, the local protocol allowed the use of steroids (methylprednisolone 2 mg/kg/day) to prevent the onset of pulmonary fibrosis in patients who maintained a PaO_2_/FiO_2_ ratio < 150 mmHg for at least 7 days of mechanical ventilation [12]. Since March 2020, the local management protocol has included tocilizumab for patients with moderate or severe ARDS and the need for mechanical ventilation (noninvasive or invasive). From the end of March 2020, all patients who received tocilizumab or high-dose steroids received standard acyclovir prophylaxis. Starting in late April 2021, remdesivir was administered to ICU patients with a disease history or onset of symptoms of less than seven days, based on the dosage of SARS-CoV-2 viremia. Selective digestive decontamination (SDD) has been introduced in the structured protocol for VAP prevention since the end of April 2021. SDD consisted of tobramycin sulfate, colistin sulfate, and amphotericin B suspension applied to the patient’s oropharynx and stomach via a nasogastric tube. Standard supportive management in the ICU did not significantly change during the study period.

### 2.2. Data Collection

Patient demographics, Sequential Organ Failure Assessment (SOFA) score, Simplified Acute Physiology Score II (SAPS II), and standard laboratory results, including coagulation and inflammatory variables, were collected upon ICU admission. In addition, the need for invasive mechanical ventilation, therapy with steroids, tocilizumab (also before ICU admission), Ganciclovir, the CMV blood reactivation, and the occurrence of new bacterial infections were collected during their ICU stay. Regarding the ICU protocol, patients were screened upon ICU admission and twice (in invasive mechanically ventilated patients) or once per week for bacterial colonization in the rectum, respiratory tract (if tracheal intubation was performed), and urinary tract.

### 2.3. CAPA Definition

Criteria for “Probable CAPA” were as follows: patient admitted to the ICU with laboratory-confirmed SARS-CoV-2 infection presenting a positive *Aspergillus* culture in bronchoalveolar lavage (BAL) or serum galactomannan (GM) Optical Density Index (ODI) > 0.5 or BAL GM ODI ≥ 1 [13,14,15,16]. These findings were considered alongside concomitant clinical and radiological signs, consistent with the definition of probable CAPA. Each patient included in the study underwent IPA screening using various methods. This screening involved monitoring serum and BAL GM, assessing *Aspergillus* growth in BAL cultures, measuring serum beta-d-glucan levels, and reviewing radiological images (chest CT). GM testing was performed on the serum and BAL samples obtained by deep tracheal aspiration using a closed aspiration system from the lower respiratory tract. Polymerase chain reaction (PCR) testing for *Aspergillus* spp. in the serum or BAL was conducted only in cases where there was significant doubt regarding IPA or when BAL GM was positive. Biomarkers were typically measured upon ICU admission and then every 4–5 days. BAL-GM testing was primarily performed in intubated patients. For each patient, the most relevant values of respiratory or serum GM and serum β-d-glucan were identified and reported, and a positive *Aspergillus* spp. culture in BAL or tracheal aspirate and a positive PCR test for *Aspergillus* spp. in serum or BAL were assessed. Patients undergoing treatment for IPA were identified, and the type of treatment was defined according to CAPA recommendations [15]. The first-line treatment was voriconazole at a loading dose of 6 mg/kg twice daily for two doses, followed by 4 mg/kg twice daily. As recommended, patients with CAPA underwent therapeutic monitoring of the drug once or twice weekly in cases of fully sensitive *Aspergillus* spp., specifically voriconazole [15]. Secondary infections were defined in line with international guidelines [17,18] and categorized as hospital-acquired pneumonia (HAP), including ventilator-associated pneumonia (VAP) and bloodstream infection (BSI). All microbiological samples were analyzed at the local microbiological and virology laboratory.

### 2.4. CMV Blood Reactivation Definition

As for the ICU protocol, patients were screened upon ICU admission and twice (in invasive mechanically ventilated patients) or once per week for CMV-DNAemia with quantitative C-reactive protein reaction in the whole blood. The CMV reactivation was set for a DNAemia > 62 UI/mL in the whole blood, the detection threshold of the method used (Abbott, Real-Time CMV, Rome, Italy).

### 2.5. Data Analysis

After initially describing the entire population, we focused on the subpopulation that developed CAPA and divided these patients into two groups: those who developed CMV blood reactivation during their ICU stay and those who did not. Categorical variables are expressed as absolute numbers and percentages, and continuous variables are expressed as medians and interquartile ranges (IQR). For comparison, the Chi-squared or Fisher’s exact test was performed for categorical variables, and the Mann–Whitney U-test was used for continuous variables. The independent association between different variables and in-hospital mortality censored at day 90 was estimated using a multivariable Cox proportional hazards regression model, including all variables associated with a *p*-value < 0.2 in the unadjusted analysis, and forcing the variable considered relevant in the model. Patients discharged from the hospital before day 90 were considered to have survived. Kaplan–Meier curves were performed to estimate the crude association between CMV blood reactivation and 90-day mortality in the subpopulation of patients developing CAPA. SPSS (version 22.0; SPSS Inc., Chicago, IL, USA) was used to perform statistical analyses.

## 3. Results

In total, 579 patients were included in the analysis. Of these, 96 (16.6%) patients developed CAPA, whereas the remaining 483 did not. Table 1 presents the demographic and baseline characteristics of the included patients, as well as comparisons between those who developed CAPA and those who did not. Patients who developed CAPA exhibited significantly higher severity scores and lower lymphocyte counts upon ICU admission than controls. The use of steroid therapy and tocilizumab was similar between the two groups. A significantly higher proportion of patients who developed CAPA underwent SDD during their ICU stay. Furthermore, 87.5% of patients with CAPA required invasive mechanical ventilation (IMV) (*p* < 0.001).

We then conducted an analysis comparing patients with CMV blood reactivation during their ICU stay to those without CMV reactivation in the CAPA population (n = 96). Of the 96 patients with CAPA, 40 (41.7%) developed CMV reactivation. The median time from ICU admission to CMV reactivation was 18 days (IQR 7–27), with higher peaks of CMV DNA load in the CAPA population than in patients without CAPA (Table S1). The baseline characteristics and main interventions in the comparison of patients with and without CMV blood reactivation were similar, as Table 2 shows. Notably, patients with CAPA who developed CMV blood reactivation had a significantly higher rate (97.5%) of IMV (*p* = 0.012).

**Figure 1:**
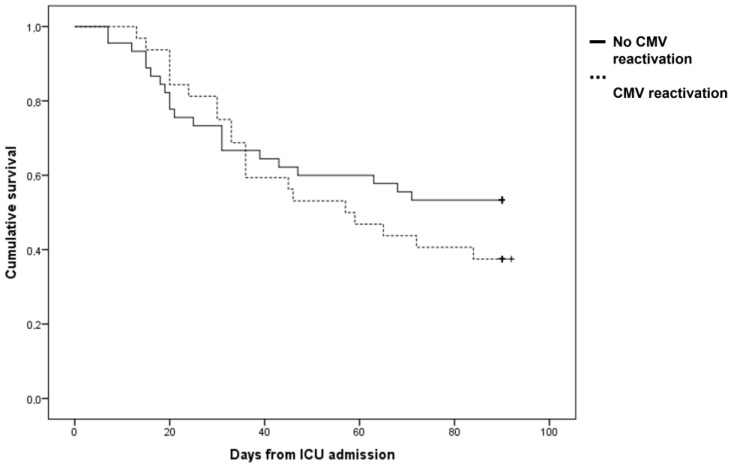
Kaplan–Meier curve for cumulative 90-day survival in CAPA population developing CMV blood reactivation or not.

The 90-day mortality rates were 62.5% in the CAPA+CMV group and 48.2% in the CAPA and no CMV group (*p* = 0.166) (Table 3). A longer ICU LOS was observed in the CAPA+CMV group (*p* < 0.001). In the CAPA+CMV group, 77.5% of patients developed a secondary bacterial infection during their ICU stay, compared to 53.6% of patients in the CAPA and no CMV group (*p* = 0.016) (Table 3). The median time to secondary infection occurrence in the CAPA population was 11 days (IQR 6–17). The CAPA+CMV group had lower ventilation-free days compared to the CAPA and no CMV group (*p* = 0.020). Moreover, CMV reactivation occurred more frequently in patients who developed CAPA later during their ICU stay (median 9.5 days vs. 2 days, *p* < 0.001). Kaplan–Meier curves demonstrated no difference in cumulative survival between CAPA populations with or without CMV reactivation (Long Rank = 0.335) (Figure 1).

The Cox regression multivariate analysis for the comparison of CAPA patients with or without CMV blood reactivation at day 90 indicated that only a previous history of immunosuppression increased the risk of CMV blood reactivation censored at day 90 (HR 2.33, 95% C.I. 1.21–4.48, *p* = 0.011). The use of tocilizumab did not correlate with the adjusted risk of CMV reactivation in the CAPA population (*p* = 0.951) (Table 4). 

## 4. Discussion

Our large, single-center observational study investigated the incidence of CAPA in critically ill patients admitted to the ICU during a four-wave period of COVID-19 and established an association between this condition and CMV reactivation. In the initial analysis, critically ill patients who developed CAPA tended to be older and presented with more severe illness upon ICU admission. SDD was administered more frequently to patients with CAPA, although it was used in less than 15% of the entire cohort. Within the CAPA population, we conducted a sub-study to compare patients with and without CMV reactivation. CMV reactivation in previously immunocompetent critically ill patients was described as a frequent event, suggesting an underlying failure of the immune system induced by critical illness. In a recent meta-analysis performed by IDSA, CMV reactivation was demonstrated to be associated with increased mortality [19], but studies evaluating the effects of treating reactivation with Gancyclovir were not able to reduce mortality risk [20]. Similarly, with other critical illnesses, derangements of immune systems occurring during COVID-19 were related to an increased risk of viral reactivation [21,22,23].

To date, this is the largest study investigating this association, and it adds to the understanding of the relationship between CAPA and CMV reactivation in critically ill COVID-19 patients. 

CMV and IPA in immunocompromised hosts have been well-described [24,25]. However, there is limited knowledge about how these infections interact in critically ill patients, and even less is known about their association with COVID-19 [8]. Similarly, in a retrospective case–control study by Calderón-Parra et al. [8], CMV reactivation was much more frequent in patients with CAPA than in those without CAPA, with an incidence difference of >25%. However, a discordant point with respect to the aforementioned study [8] is the onset time of CMV replication. In the Calderón-Parra study [8], 9 out of 11 patients developed viral replication before the onset of CAPA; in contrast, our data revealed that CMV typically reactivated later, with a median time from ICU admission of 18 days compared to a median of 5.5 days for the onset of CAPA (Table S1). This difference in timing raises questions regarding the chronological relationship between CMV and CAPA in COVID-19 patients. The exact pathophysiological mechanisms underlying the interplay between CMV and CAPA in COVID-19 patients remain unclear. Although our data suggest that CAPA tends to occur before CMV reactivation in our cohort, further research is needed to understand the temporal and causal relationships between these two infections. Additionally, it is important to consider that the dynamics of infections can vary among individuals and that the timing of infection events may not be consistent across all patients.

In terms of ICU and 90-day mortality, no differences were detected between patients with CAPA+CMV and CAPA-noCMV, as highlighted by Calderon et al. [8] (Table 3). The role of CMV reactivation in critically ill patients is highly debated, and we have already discussed this argument in COVID-19 patients [26].

The main finding of this study is the multivariate Cox regression analysis of the factors independently associated with the development of CMV blood replication in patients with CAPA. Pre-COVID immunosuppression appeared to be independently associated with CMV reactivation in the blood (HR 2.33 [1.21–4.48]). Therefore, neither anti-cytokine immunological therapies nor the severity of COVID-19, as expressed by IMV and secondary bacterial infections, seem to play a role. During the COVID-19 waves, a discussion arose about whether the therapies to counteract the cytokine storm, when added to the immune dysfunction probably induced by SARS-CoV-2 infection, could further deteriorate the immunocompetence of COVID-19 patients [27,28]. This is particularly relevant for patients who develop CAPA, which promotes viral reactivation. However, this hypothesis is not supported by our data. Our data underscore once again that basal immunosuppression is the key point that plays a major role [6]; this finding argues for the chance of performing prophylaxis against the development of CMV in patients with basal immunosuppression. Anyway, the purpose of this manuscript was not to conjecture on prophylaxis or pre-emptive CMV treatment but to raise awareness about CAPA and CMV correlation as indicators of potential immune dysfunction indicating patients were more susceptible to developing secondary infections and experiencing longer stays in the ICU.

The strengths of our study are as follows: (a) The systematic protocol implemented to identify CAPA and CMV in patients with COVID-19 admitted to the ICU and (b) the large number of patients analyzed and the prospective methodology of the observations. However, there are several weaknesses and concerns to consider: (a) The data from three different ICUs still come from a single hospital, so we do not know if the results can be applied to other clinical settings and (b) the protocol for CAPA detection did not include systematic PCR testing for *Aspergillus* spp. in serum or BAL, which was performed only in cases of strong doubt regarding IPA, which may have underestimated the sample of CAPA.

## 5. Conclusions

In critically ill COVID-19 patients, CMV reactivation is common in those with a previous CAPA diagnosis. Basal immunosuppression before COVID-19 appeared to be the primary independent variable affecting CMV reactivation in patients with CAPA. Furthermore, the association of CAPA + CMV versus CAPA alone appears to impact their ICU length of stay and secondary bacterial infections but not mortality.

## Figures and Tables

**Table 1:** Demographics, comorbidities, severity scores, and laboratory results upon ICU admission in all patients and in patients with or without CAPA. The use of steroids during their ICU stay and tocilizumab before and during their ICU stay has also been reported.

Baseline	All Population (n = 579)	No CAPA (n = 483)	CAPA (n = 96)	*p*-Value
Sex (male; n, %)	419 (72.4%)	348 (72)	71 (74)	0.702
Age (median, IQR)	65 (56–72)	63 (55–72)	70 (63–75)	<0.001
BMI (median, IQR)	29 (26–33)	29 (26–33)	29 (26–33)	0.761
Comorbidities (n,%)	445 (76.9)	360 (74.5)	85 (88.5)	0.003
Diabetes	123 (21.3)	97 (20.2)	26 (27.1)	0.131
Chronic cardiac disease (n, %)	95 (16.5)	67 (13.9)	28 (29.2)	<0.001
Chronic respiratory disease (n, %)	62 (10.7)	53 (11.0)	9 (9.4)	0.635
Chronic renal disease (n, %)	21 (3.6)	16 (3.3)	5 (5.2)	0.369
Pre-existing immunosuppression (n, %)	92 (15.9)	66 (13.6)	26 (27.1)	0.001
Hematologic malignancies (n, %)	29 (5.0)	20 (4.1)	9 (9.4)	0.033
Cancer (n, %)	19 (3.3)	11 (2.3)	8 (8.3)	0.002
SAPSII score (median, IQR)	34 (28–39)	33 (28–38)	36 (33–43)	<0.001
D-dimer (mcg/L; median, IQR)	1470 (820–3020)	1510 (820–2850)	1325 (780–3660)	0.992
LDH (U/L; median, IQR)	823 (634–1104)	815 (635–1096)	916 (624–1239)	0.341
Leukocyte count (cells/mcl; median, IQR)	8.3 (5.9–11.2)	8.2 (5.9–10.9)	8.5 (5.5–11.7)	0.829
Lymphocyte count (cells/mcl; median, IQR)	0.7 (0.5–1.0)	0.7 (0.5–1.0)	0.6 (0.4–0.9)	0.008
Platelet count (1000/mm^3^; median, IQR)	219 (170–288)	222 (171–288)	205 (155–269)	0.182
CRP (mg/L; median, IQR)	6.3 (2.2–17.1)	6.6 (2.6–17.4)	5.6 (1.2–16.1)	0.069
PCT (ng/mL; median, IQR)	0.2 (0.1–0.5)	0.2 (0.1–0.5)	0.2 (0.1–0.6)	0.772
PaO_2_/FiO_2_ (mmHg; median, IQR)	102 (82–135)	102 (81–136)	103 (91–135)	0.347
IL-6 (pg/mL; median, IQR)	276.6 (93.3–834)	259.5 (80.0–770.3)	295.3 (114.9–1177.6)	0.170
Steroid (n, %)	533 (92.2)	441 (91.3)	92 (96.8)	0.066
Tocilizumab administration (n, %)	477 (82.4)	398 (82.4)	79 (82.3)	0.979
SDD (n, %)	83 (14.3)	60 (12.4)	23 (24.0)	0.003
Invasive mechanical ventilation (n, %)	347 (59.9)	263 (54.5)	84 (87.5)	<0.001
Waves				
1st wave 25 February–6 July 2020	102	88 (86.3)	14 (13.7)	
2nd wave 20 September 2020–13 February 2021	166	142 (85.5)	24 (14.5)	
3rd wave 14 February–30 April 2021	172	136 (79.1)	36 (20.9)	
4th wave 30 April 2021–8 May 2022	139	117 (84.2)	22 (15.8)	

**Table 2:** CMV replication-associated factors among CAPA patients with available serum CMV-DNA.

Variable	Total (n = 96)	No CMV Reactivation (n = 56)	CMV Reactivation (n = 40)	*p*-Value
Sex (male; n, %)	71 (74)	39 (69.9)	32 (80.0)	0.254
Age (median, QR)	70 (63–75)	70 (63–75)	71 (63–76)	0.663
Comorbidities (n, %)	85 (88.5)	48 (85.7)	37 (92.5)	0.303
Diabetes	26 (27.1)	15 (26.8)	11 (27.5)	0.938
Chronic cardiac disease (n, %)	28 (29.2)	19 (33.9)	9 (22.5)	0.225
Chronic respiratory disease (n, %)	9 (9.4)	4 (7.1)	5 (12.5)	0.375
Chronic renal disease (n, %)	5 (5.2)	3 (5.4)	2 (5.0)	0.938
Pre-existing immunosuppression (n, %)	26 (27.1)	10 (17.9)	16 (40.0)	0.016
Hematologic malignancies (n, %)	9 (9.4)	5 (8.9)	4 (10.0)	0.895
Cancer (n, %)	8 (8.3)	3 (5.4)	5 (12.5)	0.212
SOFA (median, IQR)	4 (3–6)	4 (3–6)	5 (4–6)	0.158
SAPSII score (median, IQR)	36 (33–43)	36 (33–42)	37 (32–44)	0.519
D-dimer (mcg/L; median, IQR)	1325 (780–3660)	1360 (760–3355)	1305 (800–4866)	0.994
LDH (U/L; median, IQR)	916 (624–1239)	991 (697–1264)	788 (532–1104)	0.065
Leukocyte count (cells/mcl; median, IQR)	8.5 (5.5–11.7)	8.7 (5.6–11.6)	8.3 (5.4–15.8)	0.749
Lymphocyte count (cells/mcl; median, IQR)	0.6 (0.4–0.9)	0.6 (0.4–0.8)	0.6 (0.4–0.9)	0.624
Platelet count (1000/mm^3^; median, IQR)	205 (155–269)	219 (155–289)	198 (153–255)	0.205
CRP (mg/L; median, IQR)	5.6 (1.2–16.1)	4.6 (1.4–16.9)	6.0 (1.1–14.7)	0.649
PCT T0 (ng/mL; median, IQR)	0.2 (0.1–0.6)	0.2 (0.1–0.5)	0.2 (0.1–0.6)	0.777
PaO_2_/FiO_2_ (mmHg; median, IQR)	103 (91–135)	107 (92–142)	101 (91–129)	0.483
IL6 (pg/mL; median, IQR)	295.3 (114.9–1177.6)	385.6 (194.1–1491.0)	165.2 (95.9–875.0)	0.069
BMI (median, IQR)	29 (26–33)	29 (26–33)	29 (27–33)	0.642
Steroid (n, %)	92 (96.8)	52 (94.5)	40 (100)	0.133
Tocilizumab administration (n, %)	79 (82.3)	47 (83.9)	32 (80.0)	0.619
SDD (n, %)	23 (24.0)	14 (25.0)	9 (22.5)	0.777
Invasive mechanical ventilation (n, %)	84 (87.5)	45 (80.4)	39 (97.5)	0.012
Waves				
1st wave 25 February–6 July 2020	14 (13.7)	8 (14.3)	6 (15.0)	0.969
2nd wave 20 September 2020–13 February 2021	24 (14.5)	14 (25.0)	10 (25.0)	
3rd wave 14 February–30 April 2021	36 (20.9)	22 (39.3)	14 (35.0)	
4th wave 30 April 2021–8 May 2022	22 (15.8)	12 (21.4)	10 (25.0)	

**Table 3:** Main outcomes among CAPA patients with available serum CMV DNA compared to those with CMV blood reactivation.

OUTCOME	Total (n = 96)	No CMV Reactivation (n = 56)	CMV Reactivation (n = 40)	*p*-Value
90-day mortality (n, %)	52 (54.2%)	27 (48.2)	25 (62.5)	0.166
ICU mortality (n, %)	48 (50%)	24 (42.9)	24 (60.0)	0.098
ICU length of stay (days; median, IQR)	19 (8–39)	12 (6–23)	35 (20–59)	<0.001
Invasive mechanical ventilation-free days at day 60 (days; median, IQR)	9 (1–48)	13 (1–56)	7 (0–15)	0.020
Mechanical ventilation-free days at day 60 (days; median, IQR)	0 (0–35)	0 (0–53)	0 (0–0)	0.001
Secondary bacterial infection (n, %)	61 (63.5%)	30 (53.6)	31 (77.5)	0.016
Bacteremia (n, %)	24 (25%)	12 (21.4)	12 (30)	0.339
Pneumonia (n, %)	49 (51%)	25 (44.6)	24 (60)	0.914
Time to CAPA occurrence (days; median, IQR)	5.5 (1.0–12.0)	2 (1–7.5)	9.5 (5–20.5)	<0.001
Time to secondary bacterial infection (days; median, IQR)	11 (6–17)	10 (8–12)	19 (6–29)	0.667

**Table 4:** Cox regression analysis of factors independently associated with CMV blood reactivation in the population of patients who developed CAPA censored at day 90.

	CMV Blood Reactivation at Day 90 (n = 40)	No CMV Blood Reactivation at Day 90 (n =56)	Unadjusted HR (95% CI);	*p*-Value	Adjusted HR (95% CI);	*p*-Value
Invasive mechanical ventilation (n, %)	39 (97.5)	45 (80.4)	7.02 (0.96–51.14)	0.094	6.00 (0.74–48.75)	0.094
Secondary bacterial infection (n, %)	31 (77.55)	30 (53.6)	2.20 (1.04–4.62)	0.038	1.32 (0.60–2.93)	0.491
Tocilizumab administration (n, %)	32 (80.0)	47 (83.9)	0.74 (0.34–1.60)	0.438	0.98 (0.44–2.16)	0.951
Previous immune-suppression (n, %)	16 (40.0)	10 (17.9)	2.33 (1.24–4.40)	0.009	2.33 (1.21–4.48)	0.011

## Data Availability

The data supporting the findings of this study are available from the corresponding author upon reasonable request.

## Supplementary Materials

The following supporting information can be downloaded at: https://www.mdpi.com/article/10.3390/v15112260/s1, Table S1: Measured outcomes during intensive care stay and hospital mortality in all the patients and in patients with and without CAPA.
