# Supplementary material for: Association between Pulmonary Aspergillosis and Cytomegalovirus Reactivation in Critically Ill COVID-19 Patients: A Prospective Observational Cohort Study
Source: Viruses. 2023 Nov 15;15(11):2260. doi: 10.3390/v15112260 (PMC10675747; doi:10.3390/v15112260)
Supplement: Supplementary file 1 [file viruses-15-02260-s001.zip › viruses-2684620-supplementary.pdf]

Table S1. Measured outcomes during intensive care stay and hospital mortality in all the patients and in patients with and without CAPA.

**OUTCOME**	**All population (n=579)**	**No CAPA (n=483)**	**CAPA (n=96)**	**p value**
**ICU mortality (n, %)**	157 (27,1%)	109 (22,6%)	48 (50%)	**<0.001**
**90-day mortality (n, %)**	182 (31,5%)	130 (27%)	52 (54,2%)	**<0.001**
**ICU length of stay (days; median, IQR)**	8 (4-18)	7 (4-14)	19 (8-39)	**<0.001**
**Hospital length of stay (days; median, IQR)**	23 (15-37)	22(14-35)	31 (20-54)	**<0.001**
**Mechanical ventilation-free days at day 60 (days; median, IQR)**	51 (0-57)	53 (0-58)	0 (0-35)	**<0.001**
**Time to CAPA occurrence (days; median, IQR)**	5,5 (1,0-12,0)		5,5 (1,0-12,0)	
**Secondary bacterial infection (n, %)**	200 (34, 5%)	139 (28.8%)	61 (63.5%)	**<0.001**
**Bacteremia (n, %)**	85 (14,7%)	61 (12.7%)	24 (25%)	**0.002**
**Pneumonia (n, %)**	162 (28%)	113 (23.4%)	49 (51%)	**<0.001**
**Viral reactivations (n, %)**	181 (58,8%)	128 (53,8%)	53 (75,7%)	**0.001**
**CMV reactivation (n, %)**	117 (20,6)	77 (16,3)	40 (41,7)	**<0,001**
**Peak CMV-DNA load (copies/ml; median, IQR)**	671 (194-1741)	441 (155-1374)	1077 (499-2649)	**0,015**
**Time to CMV reactivation (days; median, IQR)**	14(4-24)	13 (3-23)	18 (7-27)	**0,154**
